# A qualitative study of implementation and adaptations to Progressive Tinnitus Management (PTM) delivery

**DOI:** 10.1371/journal.pone.0196105

**Published:** 2018-05-16

**Authors:** Anaïs Tuepker, Christine Elnitsky, Summer Newell, Tara Zaugg, James A. Henry

**Affiliations:** 1 Center to Improve Veteran Involvement in Care (CIVIC), VA Portland Healthcare System, Portland, Oregon, United States of America; 2 Division of General Internal Medicine, Oregon Health & Science University, Portland, Oregon, United States of America; 3 University of North Carolina at Charlotte, Charlotte, North Carolina, United States of America; 4 National Center for Rehabilitative Auditory Research (NCRAR), VA Portland Healthcare System, Portland, Oregon, United States of America; 5 Department of Otolaryngology/Head and Neck Surgery, Oregon Health & Science University, Portland, Oregon, United States of America; Center for Healthy Start Initiative, NIGERIA

## Abstract

**Background:**

Tinnitus is a common condition, especially prevalent among military Veterans. Progressive Tinnitus Management (PTM) is an interdisciplinary, structured, stepped-care approach to providing clinical services, including teaching coping skills, to people bothered by tinnitus. PTM has been shown to be effective at reducing functional distress, but implementation of the intervention outside of a research setting has not been studied, even though dissemination is underway within the Veterans Health Administration (VHA) system in the United States. This study was designed to address a gap in knowledge of PTM clinical implementation to date, with a focus on factors facilitating or hindering implementation in VHA audiology and mental health clinic contexts, and whether implementing sites had developed intervention adaptations.

**Methods:**

Qualitative interviews were conducted with 21 audiology and mental health clinicians and service chiefs across a regional service network. Interviews were transcribed and coded using a hybrid inductive-deductive analytic approach guided by existing implementation research frameworks and then iteratively developed for emergent themes.

**Results:**

PTM prioritization was rare overall, with providers across disciplines challenged by lack of capacity for implementation, but with differences by discipline in challenges to prioritization. Where PTM was prioritized and delivered, this was facilitated by perception of unique value, provider’s own experience of tinnitus, observation/experience with PTM delivery, intervention fit with provider’s skills, and an environment with supportive leadership and adaptive reserve. PTM was frequently adapted to local contexts to address delivery challenges and diversify patient options. Adaptations included shifting from group to individual formats, reducing or combining sessions, and employing novel therapeutic approaches.

**Conclusions:**

Existing adaptations highlight the need to better understand mechanisms underlying PTM’s effectiveness, and research on the impact of adaptations on patient outcomes is an important next step. Prioritization of PTM is a key barrier to the scale up and spread of this evidence-based intervention. Developing clinician champions may facilitate dissemination, especially if accompanied by signals of systemic prioritization. Novel approaches exposing clinicians and administrators to PTM may identify and develop clinical champions. Acknowledging the potential for PTM adaptations may make delivery more feasible in the context of existing system constraints and priorities.

## Background

Tinnitus is the chronic perception of ringing, buzzing, hissing, or other noises. People who experience tinnitus typically have been exposed to loud noise that caused peripheral auditory damage, resulting in both tinnitus and hearing loss [1,2]. Military personnel are exposed to numerous hazards that are associated with tinnitus and hearing loss, with the result that tinnitus is the most prevalent service-connected disability among Veterans [3]. In Fiscal Year 2016, 1,610,911 Veterans were service-connected for tinnitus.

Progressive Tinnitus Management (PTM) is an interdisciplinary, stepped-care approach to providing tinnitus clinical services, with five levels of care [4]. Level 1 Referral is the starting point for individuals who report tinnitus to their health care provider, and involves guidelines for referring to the appropriate provider. Most patients are referred to an audiologist to receive a Level 2 Audiologic Evaluation, which involves a routine hearing assessment plus a brief assessment of tinnitus effects. Some patients are fit with hearing aids, which can mitigate effects of tinnitus [5,6]. If tinnitus is bothersome following Level 2, patients can attend Level 3 Skills Education, involving a series of sessions (group or individual) where patients are taught coping skills for tinnitus by an audiologist and a mental health (MH) provider. The audiologist teaches ways to use sound to cope with tinnitus and the MH provider teaches coping skills derived from Cognitive-Behavioral Therapy (CBT If further services are needed, a comprehensive Level 4 Interdisciplinary Evaluation is conducted by an audiologist and a psychologist, which may result in Level 5 Individualized Support. Level 5 comprises extended individualized support for patients who are the most bothered by their tinnitus. PTM is by design a collaboration between audiology and mental health providers.

PTM has been shown in a randomized controlled trial to result in significant benefit relative to a waitlist control group [7]. Results from that study suggest that PTM in clinical settings at Veterans Affairs medical centers is effective at reducing tinnitus-related functional distress, with a relatively small number of sessions of CBT. Implementation of PTM has not yet been studied, however, from an implementation science perspective concerned with identifying barriers and facilitators that are faced when evidence-based interventions are integrated into typical clinical conditions. Evidence-based interventions frequently are adapted as they are disseminated beyond the original research setting, generating debate over the importance of fidelity to the original intervention in all aspects, or whether some modifications are unavoidable, acceptable, and even desirable [8,9]. Contextual factors are also widely recognized as influential in the success of intervention dissemination and implementation within health care delivery [10–13]. The present study was designed to address a gap in qualitative knowledge of clinical implementation of PTM to date, outside of a research setting, ahead of efforts to scale up delivery of PTM across a regional care network of the Veterans Health Administration. In particular, the study sought to identify factors facilitating or hindering implementation in audiology and mental health clinic contexts, and whether implementing sites had developed adaptations to address challenges to implementation.

## Methods

We conducted interviews with 21 participants (13 audiologists, 3 psychologists, and 5 service chiefs for audiology or mental health) from across the VHA’s northwest regional service network. The interviews were conducted between August and December, 2015, by three health services researchers (AT, CE, SN) trained and experienced in qualitative data collection and analysis methods. Participants had agreed to a follow-up interview in an earlier email survey on PTM training and attitudes. The sample included providers who both did and did not currently offer PTM at their sites. Participants worked in VHA hospitals as well as outpatient clinics in suburban and rural settings across the Pacific Northwest of the United States. Ethical approval for this study was provided by the VA Portland Health Care System Institutional Review Board (IRB #3295), and all study participants gave informed consent.

The Consolidated Framework for Implementation Research (CFIR) [13] and the Promoting Action on Research Implementation in Health Services (PARIHS) [12] frameworks informed the development of a semi-structured interview guide that asked participants about domains likely to influence implementation, such as the clinic context and characteristics including staffing (inner setting), facility or system priorities and changes (outer setting) and perceptions of PTM effectiveness (evidence). Participants were also asked to describe, when applicable, their current implementation of PTM and what aspects of the intervention were easy or difficult to implement. Interviews ranged in length from approximately 15–40 minutes and were conducted over the phone. Interviews continued until the sample was exhausted and new relevant knowledge was not being obtained from participants in the later interviews. Analysis of transcripts was done by three health services researchers (AT, CE, SN) trained in qualitative analytic methods, who double-coded nine interviews using an initial coding scheme deductively developed based on the domains embedded in interview questions. Discussion of this preliminary coding led to the iterative development of a more inductive coding scheme organized around four emergent areas: influence of setting on PTM, adaptation of PTM, influences on prioritization of PTM, and patient centered care models in the context of PTM adoption. All transcripts were double coded by two researchers, and the team met regularly by telephone to resolve discrepancies in interpretation.

## Results

Two major themes were identified and explored in the analysis. A first theme identified prioritization of PTM as a key concept and identified factors that facilitated or worked against prioritization. A second theme identified adaptations to PTM in the rich context of the local environment of diverse VA settings and patient populations. Quotes from interviewees are identified in the text as being from an audiologist (AUD), mental health provider (MH) or service chief (AUD Chief, MH Chief).

### Theme 1: PTM prioritization was rare overall, but where PTM was prioritized and delivered, this was facilitated by perception of unique value, provider’s own experience of tinnitus, observation/experience with PTM delivery, intervention fit with provider’s skills, and an environment with supportive leadership and clinic adaptive reserve

In our analysis of interviews, *prioritization* developed as a key factor determining capacity for PTM implementation (Table 1). Lack of full program delivery was most often attributed to an inability to allocate resources (time, staff, and/or space) in the context of busy clinics where providers are expected to prioritize the most urgent and severe patient health issues. Insufficient time and inadequate staffing—two different framings of the same capacity shortage—were reported, with not enough time available among direct care providers and administrative staff to deliver PTM. While most providers recognized that tinnitus is highly prevalent among Veterans, mental health providers in particular often described deciding to focus on tinnitus only with a sub-set of patients: *“Yes*, *tinnitus is number one diagnosis here in the VA*, *so it is like if you were to treat everybody–[but] the thing is*, *not everyone needs to be treated*. *Most people can deal with it…People who have more mental health issues… generally have harder times adjusting to tinnitus*, *and that is where they need the [PTM] skills*.*”* (MH7). Among mental health providers, mental health challenges such as depression, anxiety, and Post Traumatic Stress Disorder (PTSD) often took precedence over tinnitus. In audiology clinics, external demands to meet performance metrics or deliver evaluations and screening required for Veterans’ compensation and pension benefits limited the time available for PTM. Demands on administrative staff related to scheduling Level 3 (group education) and Level 4 (interdisciplinary evaluation), were cited by audiologists and mental health providers alike. Human resource shortages could rarely be addressed because the underlying logic of prioritization towards urgent problems and system performance expectations worked against PTM.

**Table 1:** Prioritization barriers (illustrative quotes).

**Focus on urgent issues**:
*“Being the manager and realizing that tinnitus is not our only focus*, *I do sometimes have to make the call on when we can focus on tinnitus and buckle down and look into all this*, *or do we just try to work with the masses and make sure that we get everyone hearing aids and only work with the ones that really*, *really need it*.*”* (AUD Chief 4)
*“Sometimes you take the issues that are right in front of your face*, *and that’s what we’re doing now… in the next*, *you know six months to probably a year*, *we’ll be looking at this [PTM] area*.*”* (MH Chief 6)
**Insufficient providers/provider time**:
*“The challenge for me personally is just the time involvement because the scheduling of the patients and the extra logistical work we don’t actually get time for*. *So we’re squeezing that in in between patients or whenever we can*. *So*, *you know*, *time blocked out for the actual teaching of the class*, *but there’s no admin time at all…So that can be really challenging… So they’ve made some things a little more efficient in terms of schedules and stuff*, *but it’s still work*, *you know*.*”* (AUD 11)
*“Finding the time in my schedule to be able to do that [PTM groups] is challenging…*.*even though I have always prioritized it*.*”* (MH 7)
**Insufficient administrative staff/staff time**:
*“This particular type of a treatment really requires some administrative support*. *And we don’t have that*. *And so that ends up being on the audiologists’ time*, *which means they have to be pulled out of clinic or do it on their lunch hour*, *you know*, *to try to pull together the documents and everything*…*we don’t have that administrative support*, *which makes it hard*….*at the end of the day*, *you know*, *I’m managing a business*. *And it’s an issue; I’ve only got one person [trained to deliver PTM]*.…*everything is coordinated around her life*. *Not necessarily what’s best for the patient or the clinic*, *but it’s around what she’s available for*.*”* (AUD Chief 19)
*“We have pretty much a revolving door of MSAs [Medical Support Assistants] at the front so that does certainly lead to some challenges as far as scheduling and triaging patients…so that’s been a big challenge for us…if somebody calls and needs to talk about tinnitus*, *then [the current team MSA] knows to put them on a day that I’m here…whereas another MSA wouldn’t*.*”* (AUD 3)
**Focus on system expectations and measures**:
*“Honestly*, *the schedule is just packed with patients both treatment and comp and pension now*, *that I don’t feel like I have time to branch out into tinnitus management with mental health*. *But*, *I’ve certainly given it some thought*. *I refer a lot of Veterans to the group education*.*”* (AUD 9)
*“The culture’s not in our favor…we’ve had work added and we’re just expected to figure out a way to get it done*. *Time and efficiency; time and efficiency’s always an issue in the clinic*.*”* (AUD 11)
*“I think half of us are definitely…[wanting to] expand*. *But I would say maybe the others just feel like they’re just trying to keep up with current work load*, *and maybe get stressed at the idea of offering more services when our demand for hearing tests and hearing aids is so high*.*”* (AUD 8)

Several factors facilitated sites overcoming prioritization challenges and offering PTM. PTM’s ability to address a gap in patient care was an important consideration for some audiologists: *“Tinnitus is such an issue…where everybody seems really happy that we have something to offer patients…*. *[patients]’ll say*, *‘every other place I’ve gone has said there’s nothing we can do’”* (AUD 10). Some mental health providers also recognized this value: *“People really want it*, *they really want to have something that they can refer patients to and patients are really interested in getting something from it”* (MH 7). The importance of being able to offer patients with tinnitus some form of education on coping strategies held value even when providers were not aware of the evidence base behind PTM, though there was an assumption that it was an evidence-based intervention.

The presence of a provider champion—defined as an individual who actively advocated for PTM—also facilitated delivery. Champions developed in multiple ways. Two providers attributed their advocacy to personal experience of tinnitus; as one stated, *“unless if you’ve experienced raging tinnitus yourself…you don’t really get it”* (AUD 2). For others, advocacy for PTM developed out of experience with the program itself and being exposed initially to in-person observations of its delivery: *“going up there [to see PTM at another facility]*, *…*.*I don’t think I would sell it as hard if I hadn’t been able to see it in action before*” (AUD 15). Finding a fit between PTM and one’s strengths or preferences as a provider also helped: *“Once I observed [PTM]*, *I thought*, *oh my gosh*, *I need to get involved in this and I want to do this because it’s*, *I’ve always been heavy into counseling*, *it’s kind of my passion and strength…my case load has become much more heavy in tinnitus management…*. *I’ve kind of become the tinnitus specialist here*, *you could say”* (AUD 10).

In addition to perceived intervention value and the presence of an individual provider champion, prioritization of PTM by clinic leadership also facilitated delivery: *“Strong administrative support and having ability to put the time into it… [our] chief basically saying that… he wanted us to be involved*, *that he is wanting us to have this [PTM] is part of our training program for all of psychology*, *that is important and they are very supportive of that*. *So things like that have been ways in which they have supported us*.*”* (MH 7)

### Theme 2: PTM is being adapted to local contexts in diverse ways to address delivery challenges and diversify patient options

Participants described numerous adaptations to the PTM intervention that were occurring at the local level (Table 2). These adaptations primarily were associated with Levels 3 and 4. Audiologists and mental health providers alike adapted PTM. The most common adaptation was an audiologist delivering PTM without a collaborator in mental health services, either to co-lead Level 3 sessions or for interdisciplinary evaluation in Level 4. This adaptation was most often attributed to audiologists being unable to identify a mental health provider with time to collaborate; one audiologist also commented that patients’ needs could usually be met without mental health collaboration. A different variation was to deliver PTM with mental health, but not advertise the fact, since these providers reported experiences with patients avoiding sessions physically located in mental health or promoted as being led by mental health providers. Physical distance between audiology and mental health services was not identified as a reason for the lack of mental health involvement.

**Table 2:** PTM adaptations (illustrative quotes).

**PTM without (much) mental health collaboration**:
*“[U]p until pretty recently [I had] a person in the mental health clinic that I could refer patients specifically to*. *And*, *she had interest in*, *um*, *learning more about tinnitus and*, *kind of*, *we did some coordination with some patients*. *But…*. *she has now left the hospital*, *and I don’t have any great contacts in the mental health clinic to refer people to*, *specifically*.*”* (AUD 3)
*“I have probably only had maybe three or four people [in two years] that I’ve had to pull in a kind of more of an interdisciplinary team approach on*, *so not very many*.*”* (AUD 12)
**Level 3 as individual, not group, intervention**:
*“We stopped doing the group*. *We had dwindling numbers and so we’ve really ended up following these people individually…*.*every time we ran a group*, *I had multiple people talk about how much they appreciated the information*. *So it is a toss-up because I think that…*.*the patients oftentimes want to be seen individually in the clinic*. *It’s what they know and it makes sense to them and they think they’re getting better care*.*”* (AUD 3)
*“Some folks due to behavioral health issues*, *for example*, *don’t do well in group settings*. *And so*, *you know for them*, *the group was more of a turn off and so they didn’t get as much out of it*, *and so then I would meet with them one-on-one*.*”* (AUD 12)
*“You know*, *folks who live… far away… they usually make the call that that’s just too hard*, *and ‘I can’t come five weeks in a row*.*’ So*, *then I work with them individually*. *And*, *I’ll even sit down and do the PowerPoints with them individually and provide them with the workbook*. *And*, *we have DVDs now that go through the class*, *kind of talk-show style*. *Um*, *so*, *you know*, *I’ll provide people with those things when they can’t*, *when they can’t make it for whatever reason*.*”* (AUD 10)
*“We don’t do that [group session] anymore*. *We had problems with patients not showing*, *difficulty or actually taking time to keep a list and then blocking a slot and then the patients not showing up…*.*that also caused difficulty finding locations…*. *We kind of decided to move it to a more*, *‘if a patient needs it*, *we’ll put them in a slot’…no group appointments…*.*we just make sure all of the providers are educated enough to give the basic tinnitus counselling*.*”* (AUD Chief 4)
**Combining Level 3 sessions**:
*“[P]art of it is my own schedule constraints…I’ve just kind of collapsed the two segments into a longer 2-hour session*.*”* (MH 21)
*“[T]he doctor from Behavioral Health who comes over is pretty busy… [and] doesn’t have quite as much time flexibility as I have*. *So he is—we’re going to try combining his classes so it works a little bit better for him to see more patients over at Behavioral Health and hopefully it will work a little bit better for my traveler [patient]s…*.*I’m always nervous about changing things that have been working well… I’m hoping that by collapsing into one session we’re not going to*, *kind of*, *overwhelm people and then they’re not going to absorb it*, *but we’ll see how it goes*.*”* (AUD 12)
**Modifying therapeutic approach**:
*“I feel like we go in with the structure of PTM in mind and…that morphs from one patient to the next*. *So I definitely don’t feel like I say and do the same thing with each patient*, *even though I go into it with the same intent…how much time I have to spend on different components of the counselling is going to be very different from patient to patient*. *So I mean*, *the structure is the same*, *but I’m not sitting there with the book…it’s more conversational*, *more*, *more clinical*, *more real world*.*”* (AUD 3)
*“I don’t like the relaxation training*, *the breathing—based one*. *And I’ve gotten that same feedback from several Veterans*. *And so*, *I’ve just kind of discarded it*, *frankly …I use more of a kind of mindfulness approach*, *might be*: *“be aware of your diaphragm”… not so structured in that regard*.*”* (MH 21)
*“I feel like we go in with the structure of PTM in mind and…that morphs from one patient to the next*. *So I definitely don’t feel like I say and do the same thing with each patient*, *even though I go into it with the same intent…how much time I have to spend on different components of the counselling is going to be very different from patient to patient*. *So I mean*, *the structure is the same*, *but I’m not sitting there with the book…it’s more conversational*, *more*, *more clinical*, *more real world*.*”* (AUD 3)

A common adaptation among study participants was the delivery of Level 3 in an individual rather than group encounter. The effectiveness of PTM Level 3 has never been theorized to depend on a group dynamic, but PTM was initially developed and tested with provision of Level 3 in a group setting. This change in delivery mode was attributed to a perceived lack of patient demand (especially in small or rural sites), as well as a perceived patient preference for individual care. Another change to the delivery of Level 3 included offering it via Telehealth at one rural site, with several participants described wanting to be able to deliver Level 3 either in this way or through on-line applications. A few providers consolidated multiple Level 3 sessions into a smaller number of longer sessions, either because this was felt to make it easier for patients to attend or because it was easier for providers to schedule.

While audiologists more often commented on and seemed to lead changes to the format of PTM delivery, one mental health provider described modifying the underlying therapeutic approach to Acceptance and Commitment Therapy (ACT) rather than CBT, and another described substituting a different relaxation technique from that used in the PTM materials. The PTM workbooks were widely used as a patient resource, regardless of the level of PTM implementation, but several providers minimized time spent on workbook or video material that they felt was repetitive or out of date. Additionally, several providers would have used the workbook, but were no longer able to obtain copies. Both audiologists and mental health providers indicated that, as they became comfortable with PTM, they tailored delivery more to the individual patient’s needs and preferences.

## Conclusions

In this qualitative study, PTM implementation was found to be highly influenced by the extent to which implementing individuals and sites prioritized the intervention, and that multiple factors could enhance or discourage prioritization. Our interview guide did not explicitly ask about prioritization of PTM, yet this dynamic came to the forefront of participants’ comments repeatedly. Disciplinary priorities mattered and differed between audiology and mental health. Within the CFIR model used to frame our investigation of PTM implementation, “relative priority” is a construct nested within the inner setting. Our results suggest however that relative priority spans the inner and outer settings, since individuals’ and clinics’ desire to prioritize PTM was influenced by a provider’s or clinic’s perceived core focus but also tempered by perceived demands imposed from above. Both the CFIR and PARiHS models stress the perceived strength of the evidence base as a factor in implementation success. Interestingly, awareness of the evidence base for PTM did not emerge as an important factor either facilitating or hindering implementation; while providers may have been aware of the existing evidence base to support PTM, other factors such as staffing capacity and broader facility priorities for performance improvement, assumed greater importance, in the accounts they shared with us, in deciding whether or not to implement or expand PTM delivery.

Prioritization as a process allowed us to make sense of the convergence of intervention, provider, and clinic/context characteristics that determined whether PTM was offered or not. Prioritization can be seen as the intermediate goal of “finding a fit” between PTM and one or more provider characteristics (such as existing skills and interests) and system expectations (which might also be described as compatibility), which then makes PTM implementation and spread more likely. Among the factors identified as affecting prioritization, exposing clinicians and administrators to PTM may be the most approachable target for improving prioritization. It may not be practical to support clinicians and administrators to visit sites using PTM, but video recordings of pertinent portions of PTM sessions or online toolkits sharing clinical interactions at sites currently using PTM could improve exposure to PTM to clinicians and administrators who are unable to see PTM in person, and help address common concerns about how to efficiently target appropriate candidates. These tools could also frame PTM in ways that encourage viewers to consider identifying themselves as potential champions. Novel methods of exposure could also include personal accounts from patients who have learning coping skills for tinnitus, and from clinicians who are teaching coping skills.

Our findings suggest that adaptations of PTM on the ground are numerous, and that impacts of these adaptations are not yet being tracked. We note that our study, conducted only within the VA system, should not be generalized outside of the VA context, where different barriers to collaboration between audiologists and mental health providers, for example, may be even more salient. Cohen and colleagues [8] have observed and categorized intervention adaptations as accommodations to practice needs, patient needs, and personnel costs. We encountered the same rationales for adaptations, yet some of the adaptations reported in this study, such as delivering individual rather than group Level 3 Skills Education, were justified on different grounds in different contexts. Literature on CFIR has frequently discussed the need to identify core aspects of an intervention, and similarly the realist evaluation approach emphasizes the need to understand the mechanism of an intervention—why it works in a particular context [14]. Our finding that providers frequently adapt PTM highlights the need to understand better what mechanisms drive PTM’s effectiveness and thus which components of PTM can be modified while maintaining or enhancing positive impacts. The delivery of Level 3 in a group setting could be argued to be a matter of clinic convenience/provider availability (a practice accommodation) not core to PTM’s functioning; alternatively, therapeutic components of peer support not articulated or envisioned in PTM’s development may be a mechanism for effectiveness, especially for some patients, while others may benefit from individualized attention. Research with patients who participate in individual or group Level 3 PTM settings is indicated as a next step for understanding this mechanism.

Potentially, the most significant adaptations found by this study involved changes to the underlying therapeutic approach employed by mental health providers delivering Level 3 education. The substitution of one therapeutic philosophy for another (ACT for CBT) might be seen as a significant adaptation requiring testing in a clinical trial, but drawing the line between an adaptation and a new intervention is not always straightforward, and the ideal of full clinical trials as either necessary or efficient to measure the impacts of adaptations is being increasingly contested in dissemination and implementation research [15]. The two primary approaches to tinnitus intervention include sound-based therapy and counseling, both of which are important to consider in meeting specific patients’ needs and preferences. Systematic reviews have supported CBT as the most evidence-based intervention for tinnitus, as pointed out by the AAO-HNSF *Clinical Practice Guideline*: *Tinnitus* [16]. Our own intervention trials have supported the role of both audiologists and MH providers in providing effective tinnitus care. The evidence for including MH providers in tinnitus care thus includes a Cochrane review [17], the AAO-HNSF guidelines, and our own trials. Currently PTM is rooted in the theoretic framework of CBT because it has the strongest evidence base as an effective intervention for reducing distress from tinnitus.

However, there is research evidence for other theoretic frameworks, Acceptance and Commitment Therapy (ACT), and Mindfulness Therapy as effective approaches to reducing distress from tinnitus [18,19]. The importance of PTM adapting and fitting to both provider skills and patient preferences, which our findings suggest, would argue for a flexible approach and the development and testing of effectiveness of versions of PTM using ACT, Mindfulness Therapy or other approaches proposed by adopting clinicians. PTM is being adapted on the ground, and further studies need to investigate PTM’s underlying mechanisms for effectiveness, in particular, whether group and individual education, and the use of standardized versus tailored therapeutic approaches, work differently and have different measurable impacts on patient well-being.

As with any small qualitative study, our findings cannot be generalized to other contexts; however, as PTM was developed and is currently used mainly within the VHA system, this context is the most relevant for the subject being studied. Additionally, while we interviewed providers in diverse settings in one region of the United States, we did not include all VHA audiology and mental health services in our sample; given that our interview sample was drawn from providers who had already taken a survey about PTM, there is a bias towards providers with some awareness of PTM. Participation by mental health providers was especially limited, since many in our potential sample pool had limited experience with PTM. Different barriers and facilitators may be relevant where no prior knowledge of PTM exists. However our finding that system performance expectations and human resource shortages are key barriers to new interventions being prioritized is consistent with prior work on broader system transformation within VHA [20,21].

Prioritization of PTM is a key barrier to the scale up and spread of this evidence-based intervention in VHA facilities. Developing clinician champions, through exposure to PTM training and methods as suggested above, is likely to be important to increasing dissemination to interdisciplinary teams of audiologists and mental health providers. Effective dissemination is also likely to be aided by promoting other facilitating factors identified by this research, especially if accompanied by signals (such as mandatory training, dedicated provider and staff time) that clinics, and the systems within which they operate, recognize the potential added value of PTM in addressing the complex health needs of Veterans.

## Disclaimer

The views expressed in this article are those of the authors and do not necessarily reflect the position or policy of the Department of Veterans Affairs or the United States government.

## Supporting information

S1 File Phase 3 interview guide. (DOCX)
