# Supplementary material for: A qualitative study of implementation and adaptations to Progressive Tinnitus Management (PTM) delivery
Source: PLoS One. 2018 May 16;13(5):e0196105. doi: 10.1371/journal.pone.0196105 (PMC5955512; doi:10.1371/journal.pone.0196105)
Supplement: S1 File — (DOCX) [file pone.0196105.s001.docx]

**RRP Phase 3 Interview Questions**

As is conventional within qualitative research using semi-structured interviews for data collection, the exact wording of questions and probes will vary. The interviewer will be attentive to establishing rapport, context and previous participant answers, and will use appropriate communication styles, all of which may influence precise wording. The questions and probes listed here are representative of the general content to be explored in the interview.

1. **Sample 3—Audiology and Mental Health Chiefs/Supervisors and Clinicians**

Interview Questions: (items in parentheses and gray color indicate RE-AIM and/ or CFIR theory components or implementation outcomes of interest)

| **Study Objective** | **Interview Question** |
| --- | --- |
| O3 | 1. Tell me about your role in Audiology (or Mental Health)? Have you been involved at all with providing clinical intervention targeted specifically at helping patients improve quality of life with tinnitus? |
| O3 | 1. What has been your clinic’s experience generally with making changes in how you deliver care, or in doing quality improvement work? (readiness for change, adaptive reserve)  - Possible prompts: Is there time for innovation? Do people have positive/negative attitudes towards change in general? Exhaustion/stability in staff as factors? Risk-averse vs “early adopter” clinic culture |
| O3 | 1. How much interest do you think there is in your clinic in offering services for tinnitus? Do you know of any existing goals to adopt strategies to help patients improve quality of life with tinnitus?  - Possible prompts: Perception of patient need, How well do you think available interventions for tinnitus align with the goals and needs of the patients you see? If not, why not? (acceptability, appropriateness) Perception that available interventions are effective? (intervention evidence base) Perception that improvements in quality of life are feasible--if not, why not? (trialability, adaptability) Do you think that there is general agreement among clinicians here in how to deal with this issue, or have you observed a range of approaches? |
| O3 | 1. Describe your facility’s current approach to helping patients improve quality of life with tinnitus in as much detail as possible.  - Prompts: When applicable, get descriptions of assessment procedures used to determine if patients want/need tinnitus services, in addition to a description of the services provided after assessment. If PTM is offered, ask about implementation level by level (levels of complexity for more detailed information - Level 1 Triage/Referral (Note: The name of this level changed from Triage to Referral—the interviewee may only recognize the term that was in use when he/she first learned about PTM.) - Level 2 Audiologic Evaluation - Level 3 Group Education/Skills Education (Note: The name of this level changed from Group Education to Skills Education—the interviewee may only recognize the term that was in use when he/she first learned about PTM.) - Level 4 Interdisciplinary Evaluation - Level 5 Individualized Support |
| O3 | 1. Have you worked with other services, like Mental Health or Audiology, to address patient needs for tinnitus services? What was that experience like? *If no tinnitus coordination:* Have you worked with other services to address any specific patient care coordination needs? What was that like?   (coordination/communication)  5a. Are you co-located with Mental Health/Audiology? If not, how far away are they? Do you have any shared space that either service can use for things like patient classes? (physical space, proximity) |
| O3 | 1. *If providing management currently*: What makes implementation of services for tinnitus easy for you?   *If not providing management currently*: What do you think would help make implementation of services for tinnitus feasible? (implementation facilitators: acceptability, appropriateness, costs, feasibility)   - - Prompts: Program services, policies, leadership support, human resources, geographic location   - Are there specific aspects of the PTM intervention that make it easy to implement?   - Can you share any examples of how other services (Audiology, mental health, IT, HR) have supported implementation of services for tinnitus/PTM?   - Can you share any examples of how providers and senior leaders (facility, VISN, VACO) have supported implementation of tinnitus services/PTM? (implementation)     1. If yes, how has leadership’s approach changed over time? What do you think influenced this change? |
| - O3 | 1. *If providing management currently*: What makes implementation of tinnitus services/PTM difficult for you?   *If not providing management currently*: What do you see as the barriers to implementing tinnitus services?   - Prompts: Program services, policies, leadership support, human resources, geographic location (implementation barriers: acceptability, appropriateness, costs, feasibility, space, location)   *If offering any management:* What are some things you do to overcome those difficulties? (adaptation, adaptability, fidelity)  *If offering PTM:* Are there specific aspects of the PTM intervention itself that make it difficult to implement?   - - Adaptations (adaptability) to the program or toolkit to fit patients or organization?   - Can you give me examples of how other services (Audiology, Mental health, IT) have supported provision of services for tinnitus? Coordination and communication (networks, communication) |
|  | 1. *Only if offering PTM*: Please tell me about what worked well and what did not in your efforts to implement PTM at this facility? (implementation)  - What did you do? What did others (e.g., clinic staff, mental health) do? - What would make implementation easier for your facility and patients? e.g. programs, services, policies, technologies, other resources? |
| O3 | 1. How do you see your role in helping your facility implement tinnitus services? Who do you think would need to be involved for tinnitus services to become a routine part of the patient care your clinic offers? (identifying champions) |
| O3 | 1. How do you see your role in helping other VAs similar to yours in terms of services offered for tinnitus? (implementation)  - Have you shared lessons learned w/ other VAs? How have you done this? - If not, is there anything you would share? |
| O3 | 1. Summary: Interviewer summarizes main points from interview and asks, “Does this summary sound complete?” |
| O3 | 1. Final question: Is there anything else you would like to add to help me better understand how to implement PTM in VA? |
